# Small-scale capture, transport and tank adaptation of live, medium-sized Scombrids using “Tuna Tubes”

**DOI:** 10.1186/s40064-015-1391-y

**Published:** 2015-10-13

**Authors:** Ido Bar, Luke Dutney, Peter Lee, Ryosuke Yazawa, Goro Yoshizaki, Yutaka Takeuchi, Scott Cummins, Abigail Elizur

**Affiliations:** Faculty of Science, Health, Education and Engineering, Genecology Research Centre, University of the Sunshine Coast, Maroochydore DC, QLD 4558 Australia; QDAFF, Bribie Island Research Centre, PO Box 2066, Woorim, QLD 4507 Australia; Department of Marine Biosciences, Tokyo University of Marine Science and Technology, 4-5-7 Konan, Minato-ku, Tokyo, 108-8477 Japan; Research Center for Advanced Science and Technology, Tokyo University of Marine and Science Technology, 670 Banda, Tateyama-shi, Chiba 294-0308 Japan

**Keywords:** Aquaculture, Broodstock, *Euthynnus affinis*, Live fish transport, Pelagic fish, Scombridae, Tuna tubes

## Abstract

The transport of live fish is a crucial step to establish fish culture in captivity, and is especially challenging for species that have not been commonly cultured before, therefore transport and handling methods need to be optimized and tailored. This study describes the use of tuna tubes for small-scale transport of medium-sized pelagic fish from the Scombridae family. Tuna tubes are an array of vertical tubes that hold the fish, while fresh seawater is pumped up the tubes and through the fish mouth and gills, providing oxygen and removing wastes. In this study, 19 fish were captured using rod and line and 42 % of the captured fish were transported alive in the custom-designed tuna tubes to an on-shore holding tank: five mackerel tuna (*Euthynnus affinis*) and three leaping bonito (*Cybiosarda elegans*). Out of these, just three (15.8 % of total fish) acclimatized to the tank’s condition. Based on these results, we discuss an improved design of the tuna tubes that has the potential to increase survival rates and enable a simple and low cost method of transporting of live pelagic fish.

**Electronic supplementary material:**

The online version of this article (doi:10.1186/s40064-015-1391-y) contains supplementary material, which is available to authorized users.

## Background

Capture and transport of live fish is essential for every fish-related enterprise, whether it is ornamental fish trade, recreational aquaria centres, in vivo fish research, aquaculture and more. For sustainable aquaculture of a novel species, whose lifecycle has not been closed in captivity, broodstock must first be established to spawn or produce seed for larvae production. The broodstock founding population must be captured from the wild and transferred to a holding tank or sea pen, and preferably, to a controlled-environment tank.

Capture and transport of live fish had been described thoroughly in several studies and aquaculture industry manuals. These however, focus mainly on fresh water species (Berka 1986), or marine species that are either slow moving with relatively low metabolism, such as reef and benthic species (Rimmer and Franklin 1997), or small bodied species and juvenile fish (James et al. 1988; Harmon 2009). Live transport of fast moving, medium to large bodied pelagic marine fish, especially Scombrids, poses extra challenges compared with benthic or reef fish. The main reasons being that Scombrid fish must constantly swim to avoid sinking (as they are negatively buoyant) as well as maintaining fresh oxygenated water movement through their gills for respiration, as they are ram ventilators which are unable to move their operculum to pump water on their gills when stationary (Farwell 2000). Ram ventilation means that the fish must either be allowed to swim autonomously, or that fresh sea water should be constantly pumped through the fish gills. Both options make the use of immersion anaesthetics impractical (because anesthetized fish will not swim, and the anaesthetics would be washed away by the sea water used to ventilate the fish gills). Another implication which arises from the high metabolism and constant swimming of Scombrids, is that respiratory and stress-related waste metabolites (CO_2_, ammonia, lactate, cortisol, etc.) are accumulated in the captured fish blood at higher rates (up to 3–4 times faster than in salmon for example, according to Korsmeyer and Dewar 2001). In addition, the fish are usually caught while feeding, and therefore their digestive tract might be full, which further increases stress, oxygen uptake and wastes accumulation (Berka 1986). Efficient removal of these wastes and replenishment of the taken oxygen requires higher flow rates of fresh seawater and often, dissolving supplemental oxygen.

Currently accepted methods for capture of live Scombrids focus mainly on rod and line fishing of individual fish. Once hooked, the fish are placed in round cornered transport tanks, which hold aerated seawater. The fish are then transferred to the holding tank at the research facility (Bourke et al. 1987; Sepulveda and Dickson 2000; Wexler et al. 2003). The use of commercial fishing vessels, capable of holding large transport tanks (larger than 5000 L), permits holding of greater numbers of fish (10–20, average size of fish 1.8 kg), for longer periods (up to 6 h), without causing stress and damage due to deteriorating water quality (Bourke et al. 1987). Alternatively, though small scale capture and transport of live yellowfin tuna (*Thunnus albacares*) was reported to be more time consuming than using larger vessels and seine nets, it allowed careful handling of each individual fish, which resulted in up to 50 % of the fish surviving capture, handling and transport, and up to 30 % of them were healthy enough to become broodstock (Wexler et al. 2003). Reducing the time spent in the transport tank is crucial for the survival of the fish, especially when using small and medium volume transport tanks (Margulies et al. 2007).

Tuna tubes have been suggested as an alternative solution for the transport of live medium-sized Scombrids when smaller fishing vessels are used, either by necessity of reducing costs, or to allow better manoeuvrability while approaching the fish schools (McPherson 2004). Tuna tubes are comprised of several opaque round or oval cylinders with a funnel shaped bottom, ranging in material from plastic, to polyvinyl chloride (PVC), stainless steel, or aluminium, which are installed vertically on the fishing vessel’s deck. The funnel at the bottom of each tube is connected to a water system, which can provide pressurized water supply. Once caught, each individual fish is placed head first in a tube, and water is pumped through the fish mouth and gills, providing oxygen and removing wastes (Bass and Alworth 1998; McPherson 2004). This method is used primarily by recreational fishermen to hold medium-sized Scombrids as live bait for game fish such as tuna species (*Thunnus* spp.), billfish (Istiophoridae family) and more. Captured fish were reported to maintain a good, healthy condition for up to 12 h (White 2011; Rudow 2011).

Once captured, the fish must be handled as gently as possible, ensuring minimal contact with the skin. Scombrids lack the protection of large scales, and have a tendency to shed their scales when captured, which makes them more vulnerable to skin infections. When bracing the fish, for example, when heavy fish must be lifted to the boat, or when transferring the fish from the transport tank to the holding tank, it is often necessary to use PVC or vinyl slings covered with water based lubricant, which have shown to be effective and less harmful than nets or bare hands (Takashi 2014). Single barbless hooks are often used to facilitate fast hook removal. Releasing the hook is then performed using long nose pliers, a hook remover, or simply by placing the fish in the transport tank with no tension on the line, so the fish will un-hook itself.

Once transferred to the holding facility, fish face major challenges to recover from capture, handling and transport related stress and injury, such as skin abrasion, hook injuries and head collision in the transport tank walls. In addition, the fish need to adapt to the tank conditions and begin feeding on the supplied feed. To assist with recovery, the fish are usually treated upon arrival with antibiotics to prevent bacterial infections, which are more likely to occur given the fish’s compromised physical condition. Treatment options include; intra-muscular injection of oxytetracycline (OTC, 100 mg mL^−1^, to a final concentration of 0.3–0.7 mL kg body weight^−1^), submersion in sodium nifurstyrenate solution (7 ppm for 1 h), submersion in formalin solution (200 ppm, for 1 h) to remove external parasites (Wexler et al. 2003), and submersion or soaking of the feed in a commercial anti-microbial agent (Trimetox^®^ 240, 20 ppm for 3 days and 50 ppm for 20 min, respectively) (Correia et al. 2011).

To avoid starvation, the fish must be conditioned to feed on the supplied diet, usually a combination of dead mackerels (*Scomber* spp.), sardines (Clupeidae family), shrimp and squid, cut to size as needed. The addition of live food has been found to induce predation activity and to encourage feeding behaviour in some Scombrid species (Wexler et al. 2003). Overcoming handling stress and instigating feeding during the first few days in the tank seem to be the most crucial factor for the fish adaptation to captivity, and once overcome, the survival rate increases significantly.

In this paper, we describe the use of a purpose-built tuna tubes apparatus to transfer live, wild-caught, medium-sized Scombrids to a holding tank, in an attempt to establish a broodstock for research purposes.

## Methods

### Broodstock tank

**Fig. 1:**
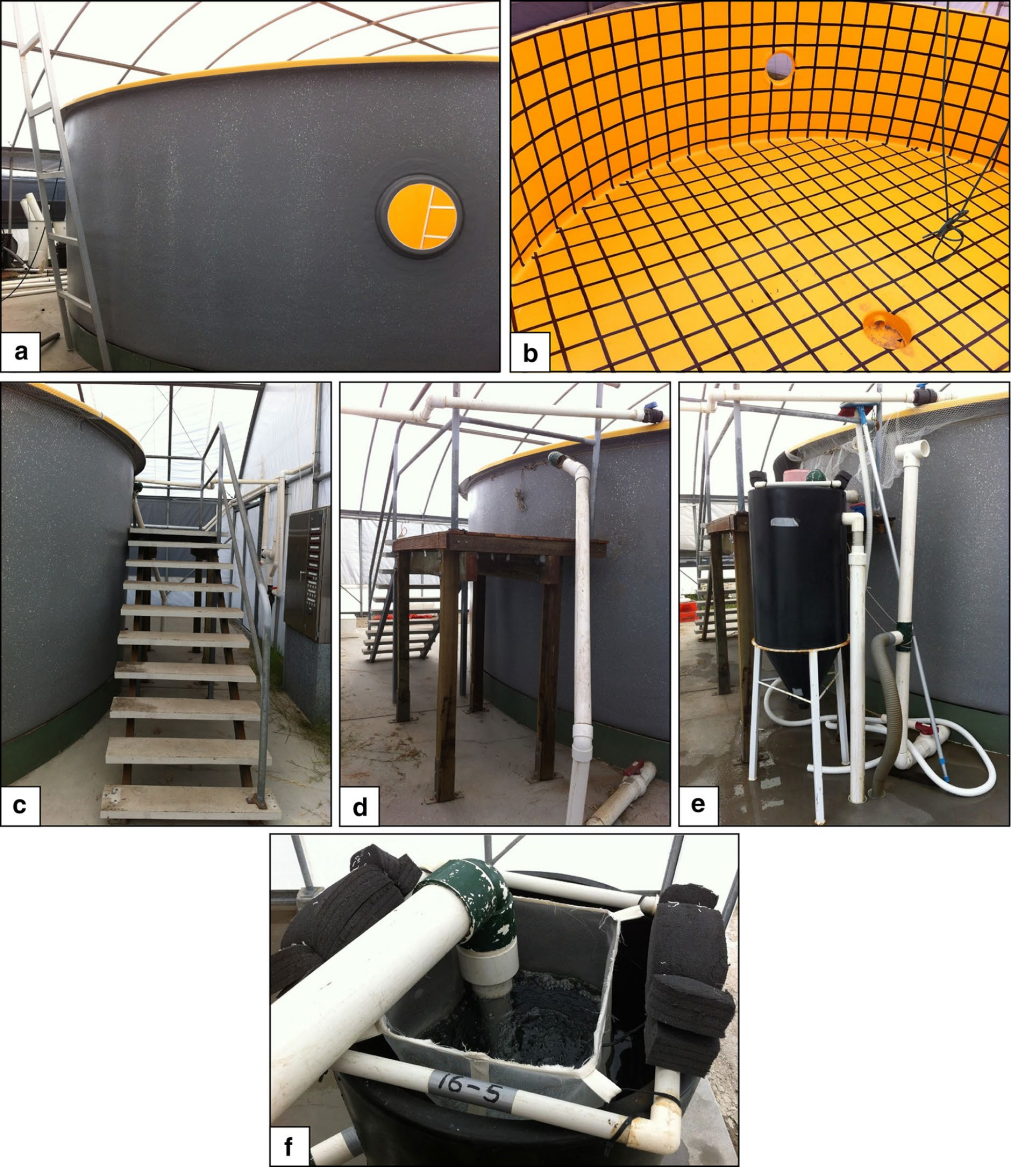
Broodstock tank at the Bribie Island Research Centre. **a**, **b** External and internal views of the purpose-built 100 m^3^ fiberglass tank (8.2 × 2.2 m). **c**, **d** Additional stairs and working platform allow easy and safe access to the tank surface. **e**, **f** Side and top view of the egg collecting apparatus and external standpipe

The rearing tank used in the study was located at the Bribie Island Research Centre (BIRC) of the Department of Agriculture, Fisheries and Forestry (DAFF) in Queensland (QLD), Australia, to accommodate the broodstock fish (Fig. 1a, b). The 100 m^3^ fibreglass reinforced plastic tank was 8.2 m in diameter, 2.2 m high. A 2 m high working platform and stairs were built to allow easy and safe access to the tank surface (Fig. 1c, d). The interior of the tank was pre-painted in the factory with an orange (Munsell Value-4) epoxy paint and black adhesive vinyl tape was applied to the interior surface in a grid pattern (0.35 × 0.35 m squares), as specified by Yazawa et al. (2011). This grid provides a visual contrast for the fish and helps them to avoid colliding into the tank walls. Additional optimization included shading of the tank to control algal growth and protect the fish from frightening and colliding into the tank walls during lighting storms. An egg collection device was installed at the water outlet, to collect floating eggs in case of spontaneous or induced spawning. An adjustable external standpipe was fitted as well to maintain minimum water level when the tank is drained, reducing the chance of accidental water drainage of the entire tank (Fig. 1e, f). The tank was supplied with filtered ocean water and continuous air inflow from the research centre’s main supply. Water quality parameters (dissolved oxygen, salinity, temperature and turbidity) in the tank were assessed on a daily basis. Cleaning of the tank and general maintenance were performed as needed.

### Small scale capture and transport of Scombrid broodstock

#### Target species

**Fig. 2:**
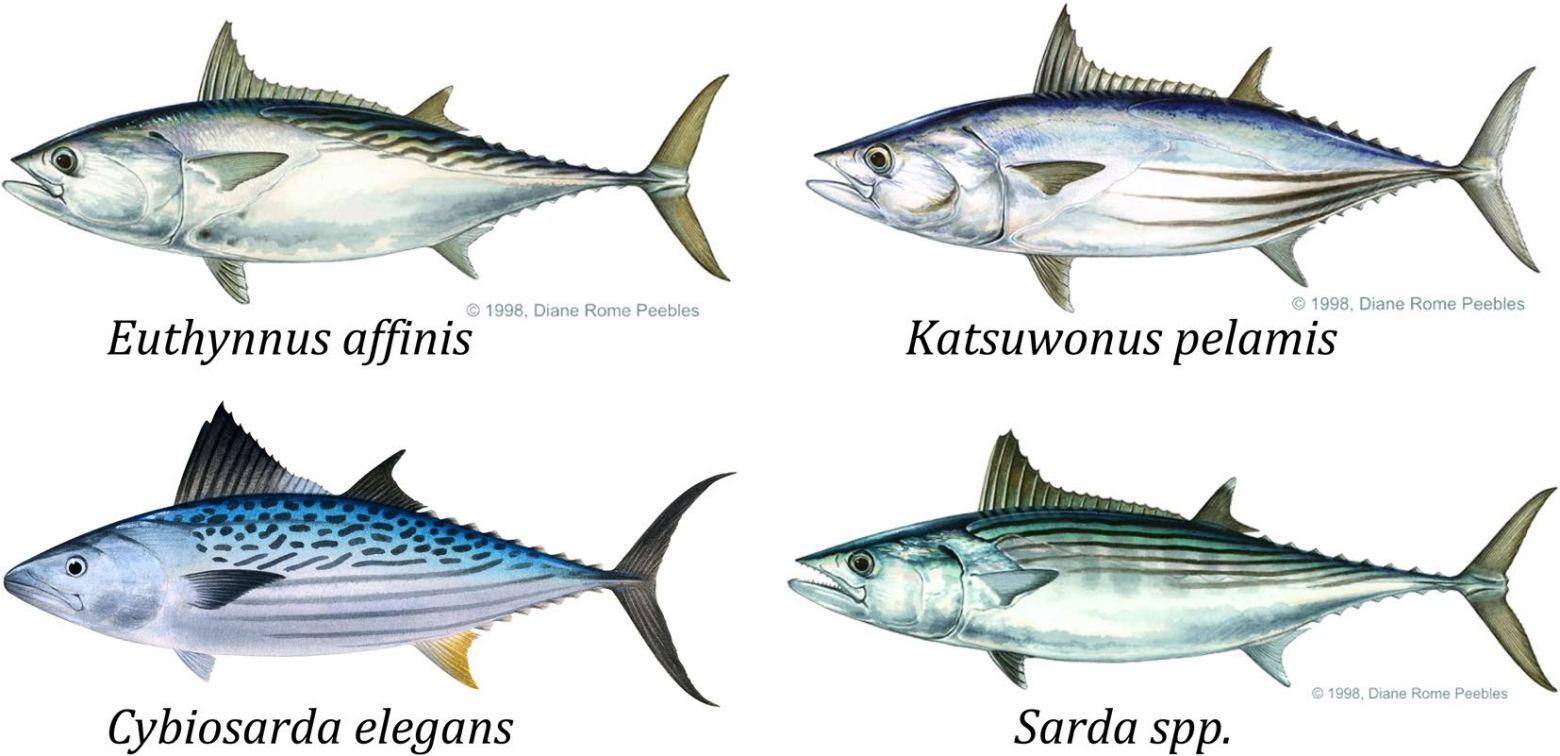
Scombrid fish species targeted as potential broodstock. Species details (from *top left*, *clockwise*): *Euthynnus affinis*—Mackerel tuna, also known as eastern little tuna or kawakawa (illustration © Diane Rome Peebles), *Katsuwonus pelamis*—Skipjack tuna (illustration © Diane Rome Peebles), *Sarda* spp.—Bonito (Australian or Pacific, illustration © Diane Rome Peebles), *Cybiosarda elegans*—Leaping bonito (illustration © R.Swainston-anima.net)

Medium-sized species of the Scombridae family that are prevalent in the Australian East coast waters were considered as potential broodstock species. These species included the mackerel tuna (also known as eastern little tuna or kawakawa, *Euthynnus affinis*), Australian/Pacific bonito (*Sarda australis/orientalis*), leaping bonito (*Cybiosarda elegans*) and skipjack tuna (*Katsuwonus pelamis*), which are presented in Fig. 2.

#### Fishing methods

Fishing for live medium sized Scombrids was performed during the Australian summer and autumn months, between December and May, when large schools of pelagic fish migrate through the Sunshine Coast region in QLD, Australia. Rod and line fishing was the method of choice, using artificial lures (mainly metal slugs, 10–20 g), that were cast towards the surface of feeding schools. Medium-light spinning rods and reels were used, spooled with a 20 lb Dacron braid line and a 3 m long 25 lb fluorocarbon leader. The lures varied in size (35–60 mm) and in colour (reflecting white/blue/silver), to specifically match the baitfish that the fish were feeding upon. Single barbless hooks and long nosed pliers were used to ensure easy and fast release of the fish into the tubes when caught and to minimise handling and potential injury to the fish. Feeding schools were visualized by presence of tern and seagull flocks that were feeding on bait fish schools. Feeding fish were approached carefully in order to avoid any disturbance to the fish and the bait; specifically, the boat was positioned upwind a feeding school and then the engine was idled, so that the boat would drift quietly towards the feeding fish. Once in casting range (10–20 m), lures were cast towards the fish and quickly retrieved. Hooked fish were brought to the surface as soon as possible, landed using a “fish friendly”, knotless rubber net (Environet, Shimano) or a PVC sling lubricated with water based gel, unhooked while maintaining minimal contact with the skin of the fish and placed head first in the tuna tubes.

#### Tuna tubes original design

**Fig. 3:**
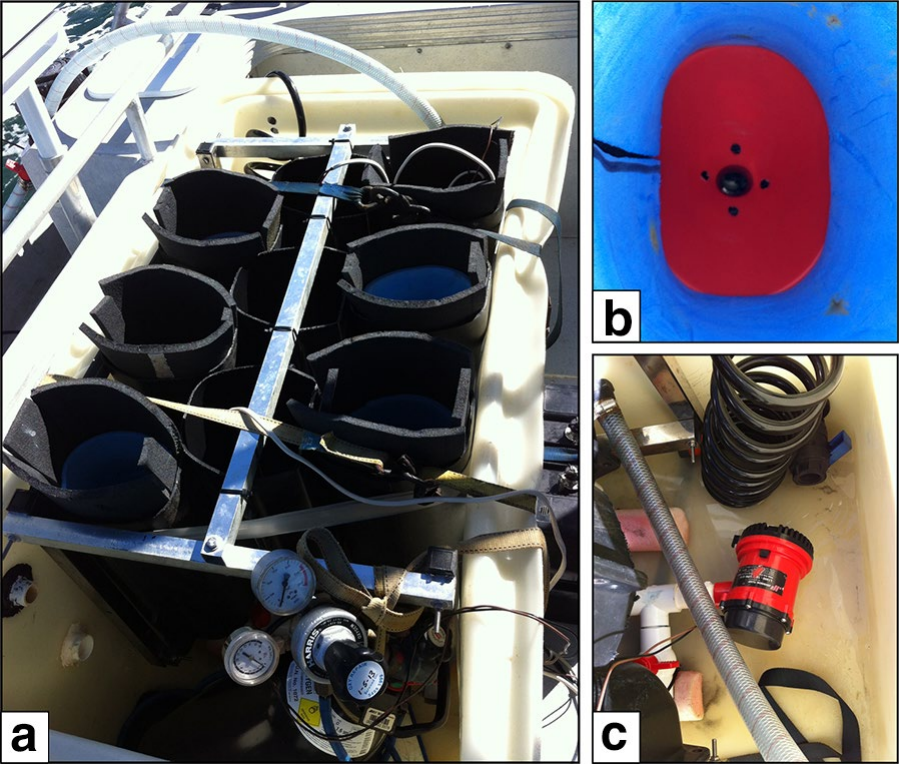
Tuna tube apparatus. **a** Six tubes attached to an aluminium frame and placed inside a 300 L tank. Note the oxygen regulator on the *bottom* of the picture and the water inlet pipes at the *top*. **b** A plastic funnel positioned at the *bottom* of the tube, directs the water over the snout and mouth of the fish. **c** One of two 1500 gallon per hour bilge pumps connected to the tubes’ water system to recirculate the water from the tank to the tubes

In order to obtain live Scombrid fish as potential broodstock, the fish had to be captured from the ocean and transported alive to the BIRC holding tank. A tuna tube unit was constructed in the BIRC workshop, based on a design described in McPherson (2004) and modified following field trials. The tuna tubes apparatus consisted of a 300 L water tank (1 × 0.6 × 0.5 m), in which six vertical oval black plastic tubes were installed (18 × 12 × 50 cm; major, minor diameters and length, respectively), held together by an aluminium frame (Fig. 3a). At the bottom of each tube, a plastic funnel was positioned and connected to the water flow system to ensure high flow of water over the fish’s mouth and through its gills.

**Fig. 4:**
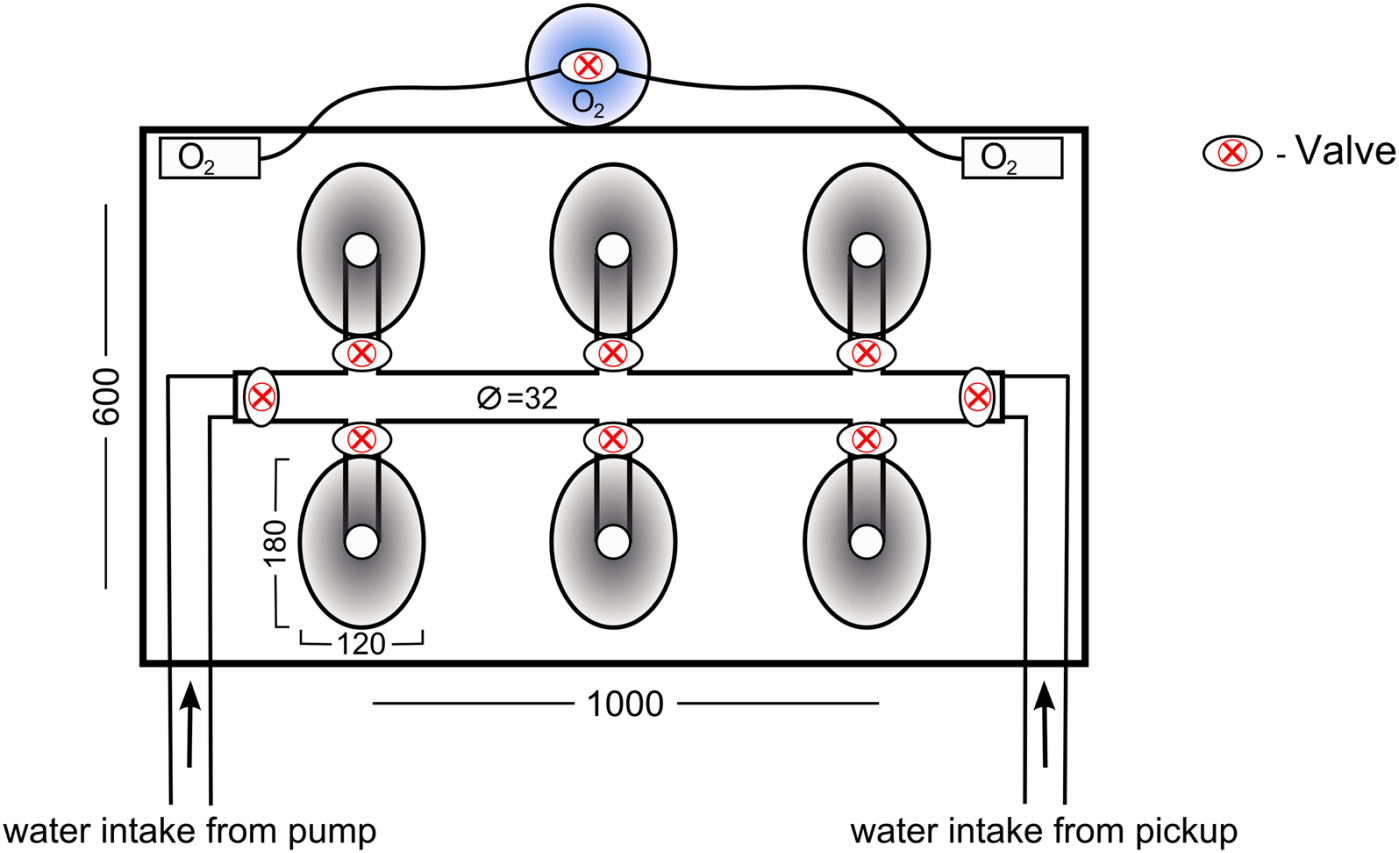
Schematic diagram of the tuna tubes transport unit (*top view*). All measurements are in millimetres

The water flow system comprised of a main water line under the tubes, with valve controlled extensions to each tube, to allow adjustable flow rate in each individual tube. Fresh ocean water intake was facilitated through a 1″ water pickup, located at the boat’s transom and lifting the water to the tuna tubes on the deck, with a head of 1 m. Additional seawater was provided through the boat’s deck pump. Two extra 1500 gph pumps (Rule, USA) were installed inside the tuna tubes tank that could be connected to the tubes’ water-flow system and recirculate the water from the tank to the tubes (Fig. 3c). This setup was used as a backup in case there was a problem with the auxiliary pump and pickup intake, or if the ocean water at a particular location was unsuitable for use, such as in a polluted marina. All the pumps were powered by a 12 V deep cycle marine battery, with a capacity of 80 ampere-hours, allowing 5–6 h of constant pumping. The water in the tank were enriched with oxygen through two gas dissolving stones and a regulated 5 L oxygen tank that was mounted on the tuna tubes tank. The tubes and their holding tank were designed to be completely portable and could be transported and mounted from boat to boat, according to the vessel availability and targeted fishing area. A schematic diagram of the tuna tubes transport unit is presented in Fig. 4.

#### Tuna tubes improved design

Between the 2012 and 2013 fishing seasons and following fish transport attempts during 2012, the tuna tubes design was modified to improve fish handling and survival. These improvements included padding of the inner tubes with soft, closed cell high-density ethylene vinyl acetate (EVA) foam, which becomes very smooth when wet, to protect the fish skin from abrasion (blue material shown in Fig. 3b). In addition, four additional holes were drilled at the bottom of the funnels to improve water flow through the tubes (Fig. 3b). Next to the water pickup, a 2200 gallon per hour (gph) bilge pump (Rule, USA) was mounted, to supply fresh water intake when the boat is stationary.

#### Fishing vessels

Two fishing vessels were used with the tuna tubes: the DAFF ‘Makaira’ (DAFF, Bribie Island, QLD), a 5.8 m long light fisheries research boat, which was launched from Spinnaker Sound Marina on Bribie Island; and the ‘Triton IV’ (South Queensland Charter services, Mooloolaba, QLD), a 7.7 m long × 2.5 m beam Reelax Capricorn Sportfisher, recreational fishing charter boat, equipped with a four cylinders, 230 hp diesel Yanmar inboard engine, which was launched from Mooloolaba Marina (Table 1).

**Table 1:** Fishing vessels used to capture live Scombrid fish

Fishing vessel	Departure site	Boat length (m)	Transport unit
‘Makaira’, DAFF	Spinnaker Sound Marina	5.8	Tuna tubes
‘Triton IV’, South Queensland Charter services	Mooloolaba Marina	7.7	Tuna tubes

#### Fish transport to BIRC broodstock tank

**Fig. 5:**
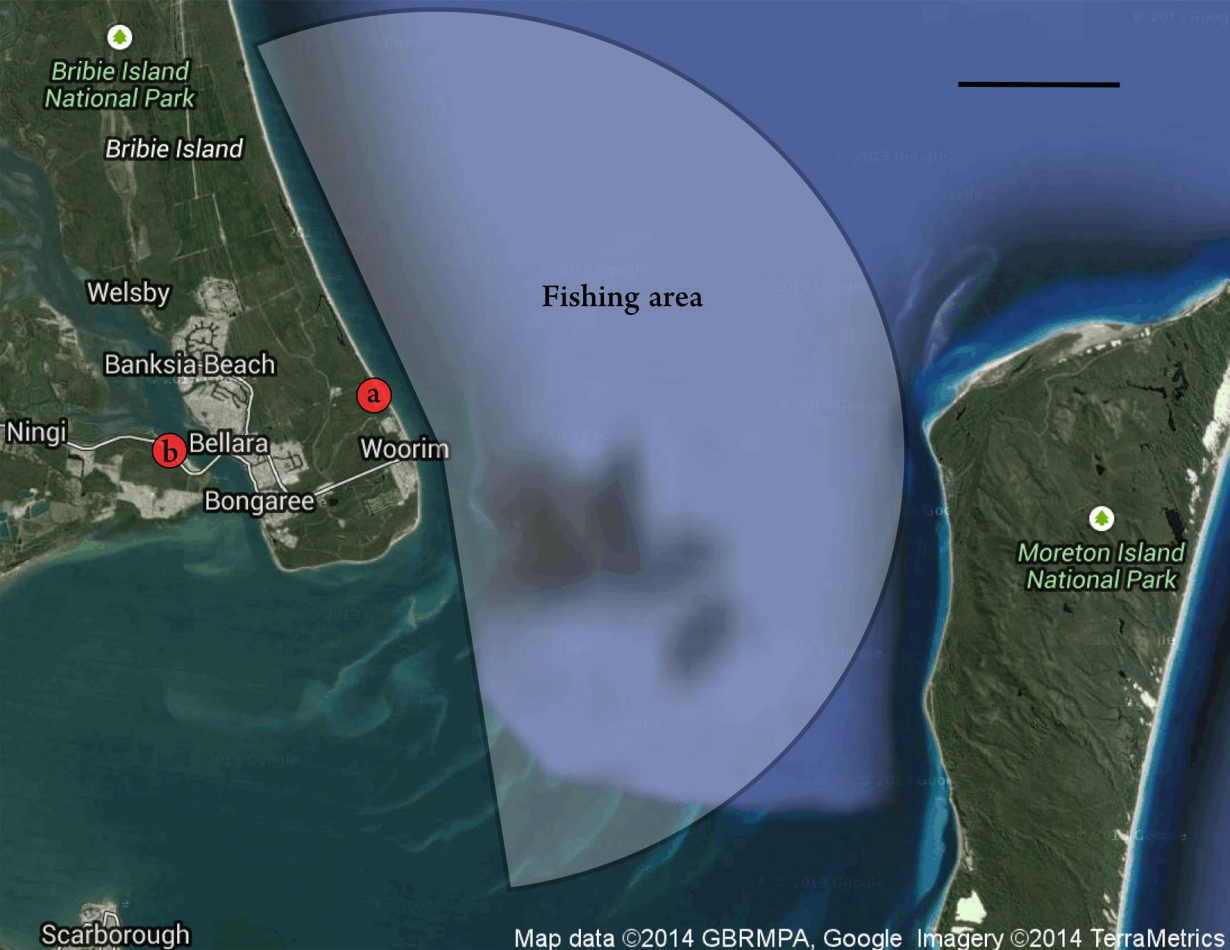
Map of fishing area around BIRC, at south-east Queensland, Australia. *Markers* on map show points of interest: **a** Bribie Island Research Centre (BIRC). **b** Spinnaker Sound Marina. *White shaded area* shows fishing grounds covered during trips. Centre of map at coordinates S27°05ʹ06ʺ, E153°16ʹ00ʺ; *scale bar* on map is equivalent to 5 km

Within 1 h of the capture of the first fish, the boat set ashore to a landing site as proximate as possible to the BIRC, to reduce to a minimum the time the fish were kept in the transport unit. When surf conditions allowed (i.e. swell height below 1 m), the boat approached the beach closest to the research centre (Woorim Beach on Bribie Island, Fig. 5a), located within a maximum of 1 h boat ride from the fishing grounds. A team member waited at the beach with a Recreational Off-highway Vehicle (ROV) and a fish transport trailer, with a 1000 L oxygenated seawater holding tank. The pumps were disconnected from the tubes, which were unloaded from the boat and quickly loaded on the ROV and then driven up to the trailer. The tubes were transferred to the tank on the transport trailer and the pumps were re-installed and connected to a battery to pump freshly oxygenated seawater over the fish. The trailer was then driven to BIRC, where the tubes were detached again from the pumps and manually transferred and submerged into the assigned 100 m^3^ broodstock tank to release the fish (Additional file 1: Video S1; Bar 2013). Overall, the transportation from the boat to the fish tank took less than 15 min, out of which the fish were in the tubes without active water circulation for a maximum of 2 min. When surf conditions did not allow for an open beach approach, the fish were transferred from the boat to the transport trailer at the Spinnaker Sound Marina, which added an additional 1.5 h to the total transport time until the fish were placed in the designated broodstock tank in BIRC. Fish that did not survive the transport to BIRC were dissected and examined to determine their sex and gonad developmental stage. Gonado-somatic index (GSI) was calculated as the percentage of gonad weight from the total body weight.

In the tank, the fish were fed on thawed bluebait (50-80 mm long, Pacific Harvest pty., Nambour, QLD) and whitebait (40–60 mm long, Pacific Harvest pty., Nambour, QLD) injected with antibiotics (OTC, 50–80 mg kg body weight^−1^ day^−1^, for 10 days) and vitamin premix (as specified by Sim et al. 2005). The tank was cleaned and the sump was drained on a regular basis to remove excretions and excess feed that had not been eaten.

### Animal ethics

All experimental procedures with live fish were performed in accordance with the University of the Sunshine Coast and DAFF Animal Ethics Committee guidelines (approval numbers AN/A/11/58 and CA 2011/10/561, respectively), following the Australian Code for the Care and Use of Animals for Scientific Purposes (NHMRC 2013).

## Results

### Fishing and transport of wild Scombrid species

A total of 14 fishing trips took place to source potential broodstock of the listed target species (medium-sized Scombrids, Fig. 1). The fishing vessels used in these fishing trips were described in the methods and are outlined in Table 1. Details of each trip and captured fish are presented in Table 2.

**Table 2:** Details of fishing trips conducted to capture broodstock of Scombrid fish species

No.	Date	Vessel	Fish species^a^ captured	Water temp^b^ (°C)	Comments
1	19/12/2011	‘Makaira’	0	24.1	Not available (NA)
2	11/01/2012	‘Makaira’	0	24.6	NA
3	20/02/2012	‘Triton IV’	0	26.5	NA
4	01/03/2012	‘Triton IV’	5 mackerel tuna, 1 skipjack tuna	26.5	None survived transport
5	24/04/2012	‘Triton IV’	0	24.4	NA
6	20/05/2012	‘Triton IV’	0	21.4	NA
7	18/04/2013	‘Makaira’	4 mackerel tuna	23.7	Two mackerel tuna survived for 11 months
8	26/04/2013	‘Makaira’	4 mackerel tuna	25.7	None survived transport
9	14/05/2013	‘Makaira’	2 mackerel tuna, 3 leaping bonito	24.1	One leaping bonito survived for 6 months
10	17/05/2013	‘Makaira’	0	24.1	NA
11	24/05/2013	‘Makaira’	0	24.1	NA
12	07/06/2013	‘Makaira’	0	23.5	NA
13	26/06/2013	‘Makaira’	0	22.5	NA
14	30/07/2013	‘Makaira’	0	21.9	NA

^a^Scientific names of species listed: mackerel tuna (*Euthynnus affinis*), skipjack tuna (*Katsuwonus pelamis*), leaping bonito (*Cybiosarda elegans*)

^b^Water temp data collected from the Integrated Marine Observing System (IMOS 2013), as recorded in Moreton Bay as close as possible to the date and time of the trip

**Fig. 6:**
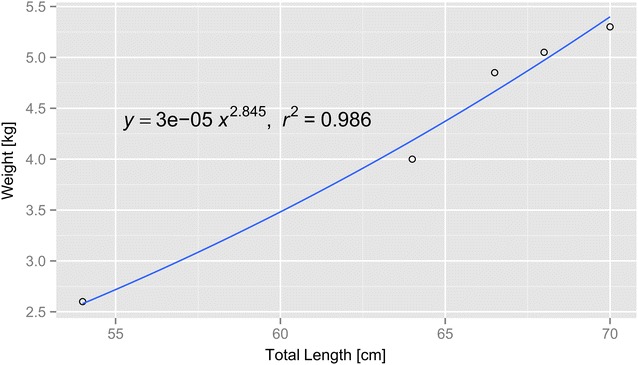
Mackerel tuna (*Euthynnus affinis*) length (measured as total length, TL) to weight relationship, as determined from caught fish

Six fish were caught during the fourth fishing trip (March 01, 2012), within 1.5 h, between 7:35 am and 8:50 am. However, the fish were caught offshore Mooloolaba, an area located 3 h boat’s ride north of Bribie island, and none of the fish survived trip to the Spinnaker Marina boat ramp at Bribie Island. The fish were examined to establish fish weight, fish length (total length, TL) to produce length to weight relationship, and dissected to determine sex and gonad weight to calculate GSI (Table 3; Fig. 6).

**Table 3:** Fish measurements of mackerel tuna, *Euthynnus affinis*, used to determine sexual developmental stage and length to weight formula, obtained from fish caught on fishing trips

Fish	Length^a^ (cm)	Body weight (kg)	Gonad weight (g)	Remarks^b^	GSI^c^ (%)
1	54	2.6	4.26	M—immature testes	0.1638
2	64	4.0	51.36	F—maturing ovaries	1.284
3	66.5	4.85	34.12	M—dark gonads	0.7035
4	70	5.3	55.18	M—maturing testes	1.0411
5	68	5.05	41.14	M—maturing testes	0.8147
6	NA	0.78	0.42	Immature gonads	0.0538
7	NA	0.68	0.357	Immature gonads	0.0525

^a^Length was measured as total length (TL)

^b^M and F denotes male or female fish, respectively

^c^Gonadosomatic index (GSI), presented as percentage, was calculated as the ratio between gonad weight to total body weight (in grams)

As a result of the high rate of fish mortalities and inability to transport live fish in 2012, the tuna tubes design was modified to improve survival, as detailed in the “Methods” section.

**Fig. 7:**
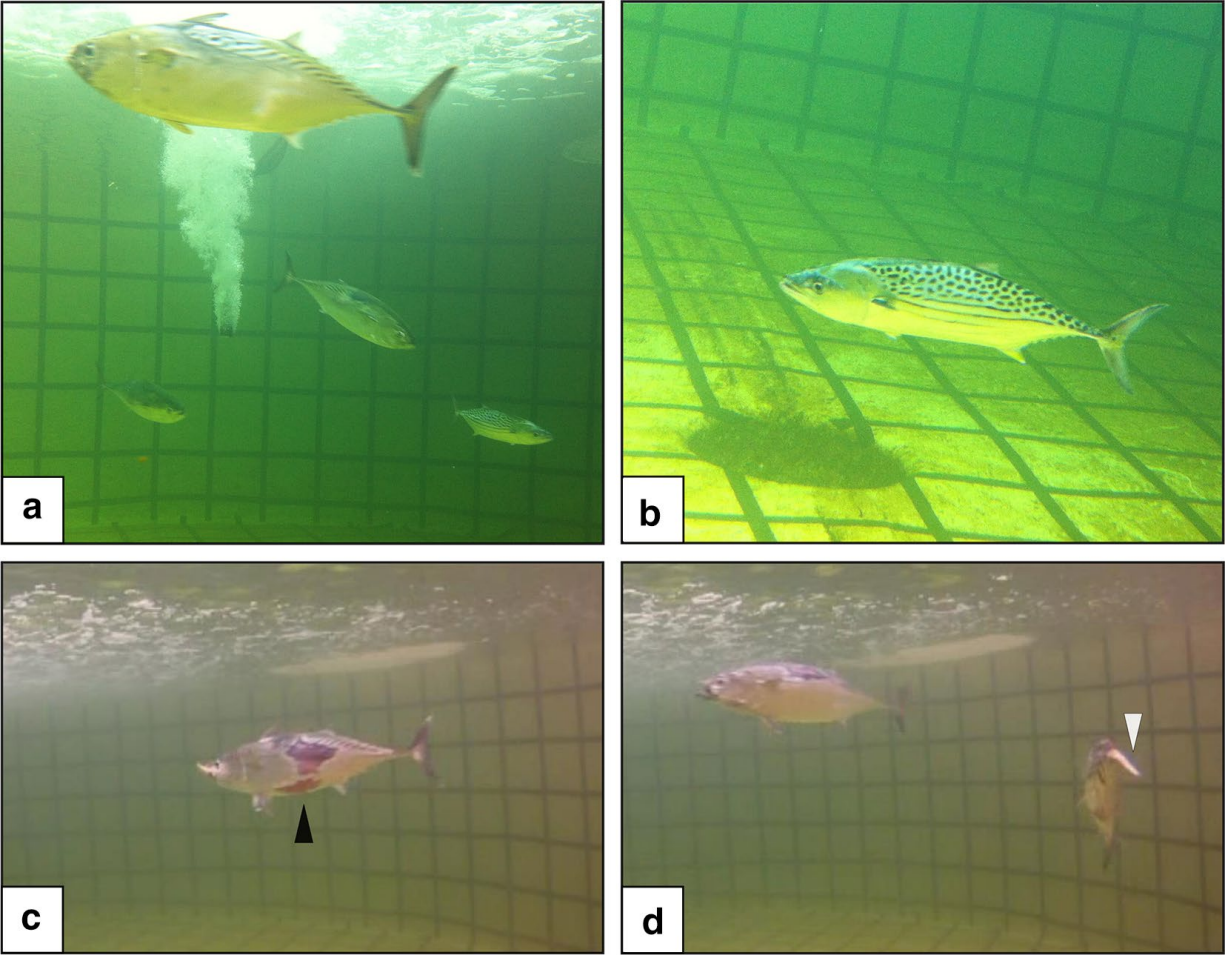
Mackerel tuna (*Euthynnus affinis*) and leaping bonito (*Cybiosarda elegans*) in the broodstock tank at Bribie Island Research Centre (BIRC). **a** Fish in tank immediately after transfer. **b** Leaping bonito. **c** Skin abrasions (marked with a *black triangle*) on mackerel tuna following transfer to onshore tank. **d** Mackerel tuna feeding on thawed whitebait, *white triangle* pointing at bait

Following those improvements, on fishing trips number 7–9, from mid-April to mid-May 2013, 8 mackerel tuna (*E. affinis*) and 3 leaping bonitos (*C. elegans*) were caught by the research team. Out of these fish, 3 mackerel tuna were successfully transferred to the holding tank on BIRC on trip number 7; another 2 mackerel tuna and 3 leaping bonitos from trip number 9 were transferred alive to the tank as well (Table 2; Fig. 7). The resident mackerel tuna seemed to interact with the newly introduced mackerel tuna, by swimming agitatedly and aggressively towards them (Additional file 1: Video S1). Live fish that were transferred into the holding tank were not weighed, to minimize handling stress and injuries, however their length and weight were estimated using the length to weight relationship (for *E. affinis*, Fig. 6) and by comparison to fish that had not survived. These fish ranged from 700 to 900 g for *E. affinis* and 400–500 g for *C. elegans*.

Some of the fish started feeding within 3 days of capture and started recovering. Fish that refused to feed did not survive. Skin abrasions were evident on the fish from the second day in the tank (Fig. 6); however, the surviving fish that were feeding recovered and their skin started healing within the first week in the tank. After acclimatization, the fish were fed ad libitum to an approximate 10 % of their body weight per day. The fish seemed to feed on the bait only as it was sinking through the upper water column in the tank and stopped once the bait had sank close to the bottom of the tank. Therefore, feed was introduced at a low rate (4–6 baitfish min^−1^, depending on the number of fish in the tank), to allow the fish to catch the bait before it became inaccessible at the bottom of the tank.

Two of the mackerel tuna that were introduced in the first batch, survived in the tank for 11 months, and one of the leaping bonitos survived in the tank for 6 months. The cause of the mortalities was unknown. Post mortem examination of one of the fish that was found in non-degraded state, revealed it was a sexually undeveloped male (GSI = 0.042 %), which grew to 1.4 kg and total length of 45.6 cm.

During fishing trips 10–14, that took place from mid-May till July 2013, no fish were spotted nor captured.

## Discussion

This study aimed at small-scale capture and transport of live medium-sized pelagic fish from the wild to a research holding tank, where they were to adapt and be maintained as broodstock. More specifically, the study assessed tuna tubes as a method of choice for transporting live medium-sized Scombrid fish, using small-scale research fishing vessels. The focus on small fishing vessels was due to a few necessities: primarily, to reduce costs and to have greater availability of research vessels, but also to improve manoeuvrability to allow beach launching and landing, enable access to small marinas, closer to the research centre, and for a stealthier approach to the feeding fish schools.

Fourteen fishing trips took place during 2012–2013 (as detailed in Table 2), from which a total of 19 fish were individually captured by using rod and reel. The captured fish were handled with maximum care and placed in custom-designed tuna tubes for transport back to the holding tank at BIRC. Out of the 19 captured fish, 5 mackerel tuna and 3 leaping bonito (making out 42.1 % of the total fish) were transported alive to the holding tank during the 2013 fish migration season. Out of these, just 3 (15.8 % of total fish) acclimatized to the tank: 2 mackerel tuna survived for 11 months and 1 leaping bonito survived for 6 months. Fish were captured in 4 out of the 14 trips, the first of them occurred in the beginning of March 2012, and the rest during a 1-month period, from mid-April to mid-May 2013. This distribution of captures demonstrates the narrow window of opportunities, when the fish are migrating through the region. The fourth fishing trip (March 01, 2012), in which six fish were easily and quickly captured at the most distant location (3 h boat ride north from BIRC), resulted in zero transport survival. Following that trip, it was concluded that to reduce transportation time, and thus increase survival chances, fishing efforts should focus on a maximum radius of 15 km, or 1.5 h boat ride from BIRC (map of fishing area presented in Fig. 5).

All the fish that survived the transport and were delivered alive to the holding tank at BIRC, weighed less than 1 kg, whereas the larger fish that were captured (at TL >60 cm and body weight >4 kg) have not survived. This pattern is likely to be related to elevated capture, handling and transport stress of the larger fish. A research studying stress levels in wild-caught rainbow trout (*Oncorhynchus mykiss*), has shown that larger fish had higher levels of plasma cortisol and lactate, known stress indicators, resulting from longer landing and handling times (Meka and McCormick 2005). Similar studies in Scombrid fish would determine whether size at capture is also positively correlated with elevated stress levels in these species.

The longest surviving fish (*E. affinis*) reached a weight of 1.4 kg after 11 months in the tank (an increase of 65 % from an approximate starting weight of 850 g), but still had a very low GSI (0.42 %), indicating it was still sexually immature. These results conform to Yazawa et al. (2015), stating that the onset of puberty in mackerel tuna is at 2 kg, therefore the captured fish required a longer fattening period in the tank. Both the narrow window of time in which mackerel tuna were captured, and the low GSI of the captured fish can be explained by the seasonal feeding and reproductive cycles of *E. affinis*. Griffiths et al. (2009) have shown that during the Australian winter (May–September), feeding activity of *E. affinis* is at its peak, while GSI is lowest; whereas in the summer months (December–February), the reproductive season, GSI peaks and feeding activity decreases. The mackerel tuna that were captured in this study were at their highest feeding activity, which assisted in detecting the schools and fishing them; however, they were at their lowest reproductive development, in preparation for the summer months. Our *E. affinis* weight-on-length relationship data that was collected and plotted (Fig. 6) was found to be consistent with known measurements for this species (Uchiyama and Kazama 2003).

Fish that were transferred alive to the holding tank at BIRC demonstrated significant skin abrasions at the dorsal and ventral widest part of the body, specifically the area bordered dorsally by the dorsal spiny and soft fins and ventrally by the pelvic and anal fins (Fig. 7c). The abrasions were not visible immediately post-introduction to the tank (Fig. 7a), but became visible within a couple of days. This pattern suggests that the skin abrasions were caused by handling and holding of the fish when de-hooking and transferring to the tubes. The time lag between handling and appearance of the skin abrasions indicate involvement of post handling bacterial infection, propagated by the removal of protective mucus and scales. Minor skin abrasion was detected at the snout of the fish as well, suggesting that the water flow through the tube was not sufficient to hold the fish back from hitting the funnel at the bottom of the tube. The abraded skin, however, healed swiftly (within 2 weeks) following antibiotics treatment (OTC, 50–80 mg kg body weight^−1^ day^−1^, for 10 days), that was administered by injection into the feed. Higher rearing temperatures were suggested to improve healing of capture and transport related injuries (Yoshizaki, personal communication).

To increase fish survival during and post-transport in the tubes, some modifications were implemented in the tuna tubes design and fish handling methods between the 2012 to 2013 seasons. These modifications aimed at reducing the handling trauma and stress levels of the captured fish, as well as reducing skin abrasions. In order to achieve those goals, the inner tubes were padded and the water flow was improved both by enhancing the water supply capabilities and changing the tube’s water inlet design, as detailed in the methods section.

The small number of fish caught, most likely due to the unpredictable nature of wild pelagic fish migration patterns, limited the opportunities to perform thorough examination of the various factors affecting the water quality parameters, fish stress levels and survival rates. However, based on the experience gained in this study, our preliminary results and available literature, a few suggestions are detailed below on how to improve the survival rate during transport in tuna tubes.

Additional measures could be taken to improve the fish survival, by maintaining low stress levels during transport. This can be achieved either by improving water quality parameters, providing oxygen and removing or neutralizing wastes, or by slowing down the fish metabolism, thereby reducing stress and reducing oxygen intake rate, wastes excretion and water acidification. Some possible methods to improve water quality parameters of the transport tank include the addition of ammonia quenchers, coupled with buffering agents to keep a stable pH and thus maintain low ammonia build-up (Correia et al. 2011). Others have suggested the use of immersion anaesthetics sedatives or cooling of the tank water by 5–10 °C as measures to slow down metabolic rates of the transported fish (Berka 1986; Rimmer and Franklin 1997; Harmon 2009). These practices however, are more applicable to closed and semi-enclosed transport tanks, which recirculates and reuse the treated water. These methods cannot be used in the case of tuna tubes, because of the high rate flow-through system they incorporate, meaning that any additives or modifiers introduced to the water will be diluted immediately.

The metabolic rate of Scombrid fish can be reduced by intra-muscular anaesthetics administered through injection of ketamine and medetomidine into the well vascularized red muscle along the midline of the body (Williams et al. 2004). This method can be used with the tuna tubes, as the sedative agent is not delivered through the water, and the sedation effect can be reversed with atipamezole when the fish are transferred to the holding tank. Yazawa et al. (2015) have shown that stress and activity levels can be reduced in mackerel tuna if the fish are kept in dark conditions during handling. Therefore, dark non-reflecting material should be used as padding to the inside of the tubes, as well as overall coverage to reduce light penetration and reflection.

Finally, to improve the overall condition of the fish, reduce the chance of physical damage (such as skin and snout abrasions), and provide better water quality, the water flow rate pumped through the tubes must be optimized. It should be strong enough to withhold the fish from reaching the funnel at the bottom of the tube, and provide sufficient water exchange to replenish taken oxygen and remove waste metabolites, but not too strong that would either push the fish out of the tube, or force it to swim to exhaustion. In order to deliver such flow rate, a designated high throughput pump should be installed with the tubes, with its independent power supply and accurate flow control. An optimized water flow rate in the tube is considered as the most important factor to maintain the captured fish in good physical condition (McPherson 2004; White 2011; Rudow 2011). Further investigation, supported by accurate water quality and flow parameters, is required to determine the optimized flow rate per species in a specific tube design; however, a good starting point is to provide a flow rate equivalent to a water current speed of one fish body length per second (Rudow 2011).

Achieving higher survival rates through the implementations of the improvements and modifications discussed earlier could enable spending longer times on the water with fish in the tubes and thus, will increase the potential fishing area that can be utilized and number of fish that can be held before returning to the research facility. In addition, it will allow transport of bigger fish that are closer to sexual maturity, and will reduce the fattening time needed in the holding tank, thus becoming more applicable for time and budget restricted projects. Tuna tubes can also be utilized as an intermediate transport system, mounted on several small fishing vessels that capture the fish and transport them to a larger “mother ship” research vessel with larger transport tanks.

## Conclusions

Upon designing and performing the fish transport trials described in this paper, it became evident that very little information is currently available regarding the live transport of pelagic fish. In fact, the only resources in the scientific literature describe usage of medium to large fishing vessels, capable of transporting big water tanks on-board (Wexler et al. 2003; Margulies et al. 2007). The only alternative that was briefly discussed for small-scale live pelagic fish transport for research operations was using tuna tubes (McPherson 2004). However, the majority of the knowledge and experience regarding the use of tuna tubes for live fish transport is withheld in the hands of recreational and semi-commercial game fishermen and is not publicly available for the research community. Considering this gap in knowledge, lack of alternatives and the complexity of live transport of fast-swimming pelagic fish, our results show, despite the limited success in capturing a large number of fish, that even basic improvements to the original tuna tubes design can improve survival rates. This study offers the knowledge and information to encourage further optimization of the tuna tubes, which should include direct measurements of stress level and water quality parameters, to provide research facilities with a cost effective and applicable method for small-scale transportation of pelagic fish.

## Additional file

10.1186/s40064-015-1391-y Transfer and feeding of captured Scombrid fish. Transport of live Scombrids (mackerel tuna and leaping bonito) using tuna tubes.Fish were transported to a holding tank at Bribie Island Research Centre (BIRC), and were maintained there for research purposes (Bar 2013). Video is accessible at https://vimeo.com/123517921.

## Abbreviations

PVC: polyvinyl chloride
OTC: oxytetracycline
BIRC: Bribie Island Research Centre
DAFF: Department of Agriculture, Fisheries and Forestry
QLD: Queensland
EVA: ethylene vinyl acetate
gph: gallon per hour
ROV: recreational off-highway vehicle
TL: total length
NA: not available
GSI: gonado-somatic index
